# Allelic Forms of Merozoite Surface Protein-3 in *Plasmodium falciparum* Isolates From Southeast of Iran

**DOI:** 10.5812/jjm.9829

**Published:** 2014-05-01

**Authors:** Adel Ebrahimzadeh, Saeed Mohammadi, Ali Jamshidi

**Affiliations:** 1 Department of Parasitology and Mycology, Zahedan University of Medical Sciences, Zahedan, IR Iran; 2 Department of Molecular Medicine, Golestan University of Medical Sciences, Gorgan, IR Iran

**Keywords:** *Plasmodium falciparum*, Merozoite surface protein 3, Plasmodium, Genetic Variation, Iran

## Abstract

**Background:**

Genetic diversity has provided *Plasmodium falciparum* with the potential capacity of avoiding the immune response, and possibly supported the natural selection of drug or vaccine-resistant parasites. Merozoite surface protein-3 (MSP-3) has been used to develop vaccines and investigate the genetic diversity regarding *P. falciparum* malaria in Iran.

**Objectives:**

The main goal of this study was to analyze the polymorphic antigen MSP-3 genes across southeast of Iran among four different districts, to identify the differences in the allele frequency and genetic diversity.

**Materials and Methods:**

Nested polymerase chain reaction amplification was used to determine polymorphisms of N-terminal region of the MSP-3 gene. A total of 85 microscopically positive *P. falciparum* infected individuals from southeast of Iran were included in this study.

**Results:**

Of the 85 confirmed *P. falciparum* samples obtained from four different districts, 72 were successfully scored for MSP-3.The MSP-3 allele classes (K1 and 3D7 types) showed comparable prevalence in all districts. Overall frequencies of K1 and 3D7 allele classes were 94.5 % for both.

**Conclusions:**

Since no study has yet looked at the extent of *P. falciparum* MSP-3 in this geographic region, these data can be helpful to support development of a vaccine based on MSP-3 against malaria. There should be a comparative analysis in different seasonal peaks to indicate the allelic polymorphism of MSP-3 over a period.

## 1. Background

Malaria is a major human health-threatening disease, resulting in approximately 300-500 million clinical cases and 1-3 million deaths each year worldwide, mainly among young children (1). Of the four species of *Plasmodium* that transmit human malaria, *Plasmodium falciparum* causes the most severe clinical manifestations of the disease and is responsible for most of the malaria morbidity and almost all of its related mortality (2). Despite enormous efforts to control and prevent malaria, multiple factors, including insecticide resistance in the mosquito vectors, lack of effective vaccines, and the emergence and rapid spread of drug-resistant strains, have been contributing to the global worsening of the malaria situation (3). Therefore, there is an urgent need for development of effective malaria vaccines (4).

However, extensive genetic diversity in natural parasites populations is a major blockage for development of an effective vaccine against human malaria parasite, since antigenic diversity limits the efficacy of the acquired protective immunity to malaria (4-7). Such extensive antigenic polymorphism intensely improves the parasite ability to invade the host’s immune system, making it difficult to evoke adequate responses against all of the antigenic variants of the parasite population (8). A true understanding about the frequencies and alterations of vaccine-candidate antigens in natural parasites populations is crucial to design a successful and effective malaria vaccine, as well as providing useful facts for interpretation of responses to the vaccine. *P. falciparum* stage-specific antigens have been characterized as vaccine candidates through molecular techniques. We analyzed the genetic diversity of merozoite surface protein 3 (MSP-3) antigen as a potential vaccine candidate.

One of the target antigens for inclusion in a malaria vaccine is *P. falciparum* MSP-3. MSP-3 is a nonintegral surface-associated protein that may be an important target for antibody-mediated protective immunity, as truncation of the MSP-3 gene reduces the parasite invasion (9). Although its function remains unknown, it has been suggested to be involved in erythrocyte binding (9, 10). *P. falciparum* MSP-3 is encoded by a single locus on chromosome 10 of the parasite (5). MSP-3 is a polymorphic antigen with a number of structural domains (6, 11). These include three blocks of four-heptads repeats of the type AXXAXXX, a hydrophilic region, and a putative leucine zipper sequence at the C-terminus (12). 

Variations among alleles of MSP-3 occur through substitutions and deletions in nonrepetitive sequences and flanking of the alanine heptad-repeat domains (13). However, there is significant conservation in parts of the molecule, particularly the alanine residues within the heptad-repeat regions, and the C-terminal half of the protein, which includes the putative leucine zipper region (14, 15). There are several sequence varieties among MSP-3 alleles, but the sequence polymorphism defines two major allele classes (K1 and 3D7), which show only limited recombination (16). The majority of both intra- and inter-allele differences are localized in the heptad-repeat region, defining the N-terminal domain (11). MSP-3 is therefore a strong vaccine candidate with limited epidemiologic data; the data needed to support its continuous development along the proposed malaria vaccine roadmap.

Iran is located in the Eastern Mediterranean region, and grouped as a low-to-moderate endemic region (17). Sistan and Baluchistan province, southeast of Iran, is the endemic area of *falciparum* malaria and considered as its oriental eco-epidemiological region (18). Malaria cases are reported during the whole year with two peaks, the first with predominant *P. vivax*, April through September, and the second with 45% to 50% *P. falciparum* infections after September (19).

## 2. Objectives

This study investigated genetic variations of the *P. falciparum* MSP-3 N-terminal domain in samples collected from four different endemic regions in southeast of Iran. To date, no study has yet looked at the extent of *P. falciparum* MSP-3 variations in this geographic region. Such data are important, because the increased frequency of simple infections in such a setting enables us to look at the allele frequency changes over the time, which might provide evidence for or against the presence of allele-specific and variant-specific immune responses.

## 3. Materials and Methods

### 3.1. Sample Collection and Study Area

**Figure 1.**
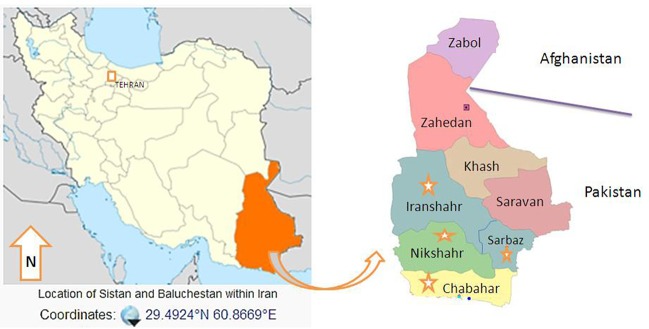
Map of the Study Area, Districts of Sistan and Baluchistan, Southeast of Iran

To characterize the genetic variations within *P. falciparum* MSP-3 in this endemic area, we initiated obtaining blood samples from *P. falciparum*-infected individuals referring to malaria centers of four different regions in Sistan and Baluchistan province, including Chabahar, Sarbaz, Iranshahr and Nikshahr (Figure 1).

A total of 85 *P. falciparum* infected blood samples used in this study were collected from patients attending the clinics and hospitals in the four study districts from March 2011 to September 2012. Residence in the regions for over 6 months, no history of antimalarial treatment for the last month, and written informed consents were required for inclusion in this study. Presence of *P. falciparum* infections in the samples were confirmed microscopically using thick and thin Giemsa-stained slides in the Department of Parasitology, Zahedan University of Medical Sciences. Venous whole blood (2 mL) was collected from each consenting patient. The samples were stored at -20°C until used for DNA extraction.

### 3.2. Extraction of P. falciparum DNA and Polymerase Chain Reaction Amplification

The DNA was extracted from the blood samples using Fermentas genomic DNA purification kit (Thermo Fisher Scientific Inc., United States). All the DNA samples were stored at –20°C before genotyping with a polymerase chain reaction (PCR).

Nested PCR was used to amplify the N-terminal region of *P. falciparum *MSP-3 gene using the external PCR primers: msp-3 (159F) and msp-3 (745R), and the internal primers: msp-3 (188F) and msp-3 (745R) (Table 1). The first and second rounds of PCR amplifications were performed in a final volume of 20 μL using AccuPower TLA PCR premix (Bioneer, Korea Republic). Cycling conditions for the first and second PCR cycles were 94°C for 5 minutes (initial denaturation), 94°C for 1 minute (denaturation), 54°C for 1 minute (annealing), and 72°C for 1 minute (extension), followed by a final extension at 72°C for 5 minutes, for a total of 25 and 35 cycles, respectively.

*P. falciparum* 3D7 (MRA-102G) and K1 (MRA-159) DNAs were purified. These strains were provided by the Malaria Research and Reference Reagent Resource Center, American Type Culture Collection (Manassas, VA) and used as positive controls during the amplification reactions. The second amplification products were directly separated by electrophoresis on a 2.0% ethidium bromide agarose gel and visualized on a transillumination imaging system (Uvitek, United Kingdom).

**Table 1.** List of Primers and Sequences.

Primer Name	Sequence Length 5’-----3’
**msp-3 (159F)**	ATGTTGCTAGTAAAGAAATTG
**msp-3 (745R)**	CATAACTAGAAGCTTCTTTTGC
**msp-3 (188F)**	ATAATCTTAACTTAAGAAATGC
**msp-3 (745 R)**	CATAACTAGAAGCTTCTTTTGC

### 3.3. Data Interpretation

Positive controls and a 1000 base pair (bp) marker (Bioneer, Korea) were used to interpret the fragments sizes. The MSP-3 K1 allele was identified as a single fragment, approximately 514 bp, and the MSP-3 3D7 allele was identified as a single fragment of approximately 448 bp (6). Mixed infections were defined by the presence of K1 and 3D7 MSP-3 alleles simultaneously. Other MSP-3 fragments of different sizes were also reported (350 bp and 500 bp).

## 4. Results

Of the 85 confirmed *P. falciparum* samples obtained from the four districts, 72 were successfully scored for MSP-3.

### 4.1. Alleles Prevalence Across the Study Area

**Figure 2.**
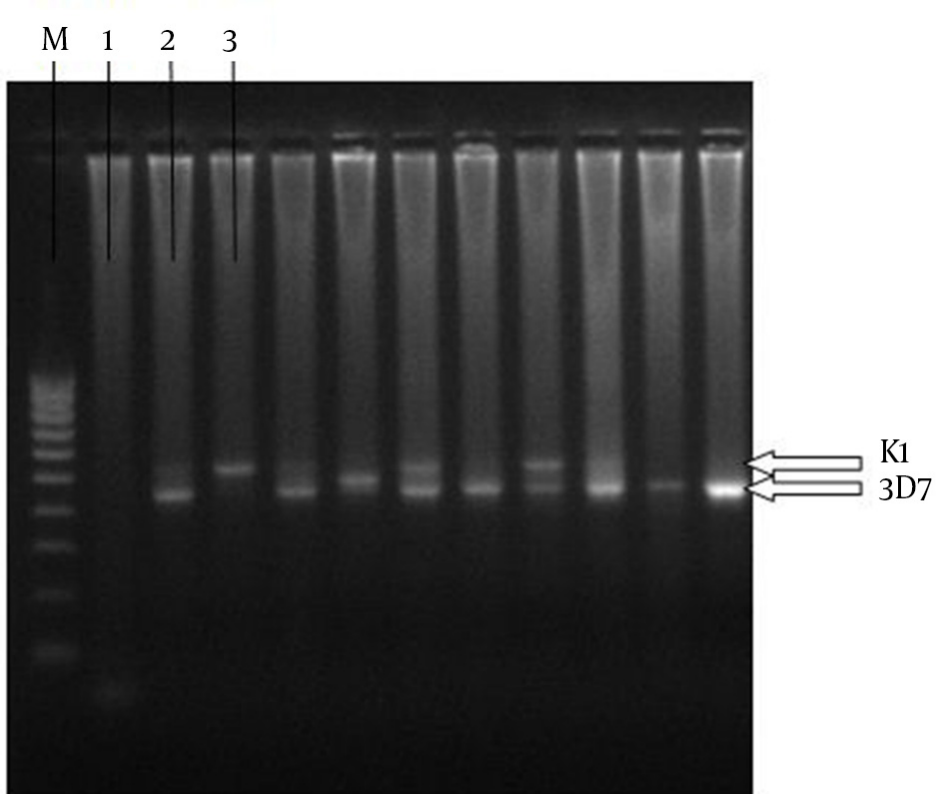
Agarose Gel Electrophoresis of the Nested PCR Amplicons From the *P. falciparum* DNAs, Extracted From Four Districts in Southeast of Iran M, DNA Marker; 1, Negative Control; 2, Positive Control of K1; 3, Positive Control of 3D7

Nested PCR was conducted to amplify and genotype *P. falciparum* MSP-3 from the *P. falciparum*-infected individuals. The primers amplified the *P. falciparum *MSP-3 N-terminal domain, where the majority of genetic diversity has been shown to occur (14) (nucleotides 117-507 in the 3D7 strain) and the allele classes was identified using agarose gel electrophoresis. The size differences confirmed that both 3D7 and K1 allele classes of *P. falciparum *MSP-3 were present in the region of the study (Figure 2).

The MSP-3 allele classes (K1 and 3D7 types) showed comparable prevalence in all the districts (Table 2). The overall frequency of K1 and 3D7 allele classes was 94.5 % for both. In contrast, the rare 350-bp allele was observed in all the districts, but was approximately three times more common in Nikshahr and Sarbaz (Table 2). The other rare msp-3 allele (the 500-bp one) appeared to be restricted to Sarbaz (prevalence = 1.9%) (Table 2). The frequencies of K1 alleles (514 bp) in Chabahar, Nikshahr, Sarbaz and Iranshahr were 53.3%, 43.6%, 44.2% and 50 %, respectively. Furthermore, the frequencies of 3D7 alleles (448 bp) in the mentioned districts were respectively 43.3 %, 48.7%, 48.1% and 45.8 %.

**Table 2.** The Merozoite Surface Protein-3 Allele Prevalence in Southeast of Iran ^a^

Allele (Length, bp)	Chabahar	Nikshahr	Sarbaz	Iranshahr
**K1 (514)**	16 (53.3)	17 (43.6)	23 (44.2)	12 (50.0)
**3D7 (448)**	13 (43.3)	19 (48.7)	25 (48.1)	11 (45.8)
**MSP3 (350)**	1 (3.4)	3 (7.7)	3 (5.8)	1 (4.2)
**MSP3 (500)**	0 (0.0)	0 (0.0)	1 (1.9)	0 (0.0)

^a^ Data are presented as No. (%).

## 5. Discussion

In this study, we used nested PCR to screen the allelic variations within the malaria vaccine candidate *P. falciparum* MSP-3 in southeast of Iran. Since nested PCR has exhibited a sensitivity and specificity of up to 94% in some tests (20), possesses a high-throughput capacity in comparison to other PCR modifications in this field of study, and is considerably more cost-efficient versus sequencing, we decided to adapt it to screen for the *P. falciparum* MSP-3 N-terminal domain variations.

Using nested PCR, we allele-typed 72 individual *P. falciparum* infections, containing the *P. falciparum* MSP-3 vaccine candidate, and found out that both 3D7 and K1 allele classes of MSP-3 were present. In addition, two other rare allele classes (500 bp and 350 bp) of MSP-3 gene were observed to some extent in some districts. This study was accomplished ignoring seasonal frequencies of each allele class.

Overall, K1 allele classes were observed in an approximately equal frequency to 3D7 allele classes. This data differed from the results reported by Jordan in a hypo-endemic transmission environment in Peruvian Amazon, with 3D7 in a higher frequency than K1 allele classes (6). Interestingly, the results reported in this study were in agreement with the results observed in western and central Africa, where K1 allele classes were in an almost equal frequency to 3D7 allele classes, reported by Issiaka Soulama (18). The possible causes of the observed changes in the allelic frequencies among geographical regions might be extraneous factors such as genetic drift. This factor is a major contributor to allele variations in small populations, which was not shown in the present study. Another hypothesis would be that differences in the genetic backgrounds among study populations may result in the selection of different MSP-3 alleles. 

In this case, the allelic frequencies might have been predicted to be stable over the time, which was not met in reality, while allele frequencies change during seasonal transmission peaks, a consequence of natural selection (8, 19). Similar allelic frequencies in this region (aside from the rare allelic forms) suggest that the distribution of the two major MSP-3 allelic forms might have been stable over time, corroborating the hypothesis that the host genetic background can influence the distribution of the EBA-175 allelic forms. Although, according to some studies, some large-scale and directional changes in allele frequencies are probable to occur over a short period of time (8). These findings confirm the presence of different polymorphic allele classes of MSP-3 in this region, while the two major classes of K1 and 3D7 were dominant with equal prevalences. Since no study has yet looked at the extent of *P. falciparum* MSP-3 in this geographic region, these data are needed to support the development of a vaccine, based on MSP-3 antigen, along the malaria vaccine road map. Furthermore, the results showed no remarkable predominance of any allele in the studied area. There should be a comparative analysis in different seasonal peaks to indicate the allelic polymorphism of MSP-3 over a period.

Recent studies have revealed the N-terminal domain of *P. falciparum *MSP-3 as a highly more immunogenic domain than the C-terminal domain (13), supporting our recommendation that the N-terminal domain should be reassessed for future vaccine developments. These data supported the hypothesis of a biologically important role for MSP-3 in the parasite development and highlighted the importance of evaluating the distribution of MSP-3 allelic forms in different geographical regions. This might provide valuable genetic information for designing an effective malaria vaccine, despite the extensive present genetic diversity.
